# The performance of common SNP arrays in assigning African mitochondrial haplogroups

**DOI:** 10.1186/s12863-021-01000-2

**Published:** 2021-10-21

**Authors:** Imke Lankheet, Mário Vicente, Chiara Barbieri, Carina Schlebusch

**Affiliations:** 1 Human Evolution, Department of Organismal Biology, Uppsala University, Norbyvägen 18C, SE-752 36 Uppsala, Sweden; 2 Centre for Palaeogenetics, Svante Arrhenius vägen 20C, SE-106 91 Stockholm, Sweden; 3 Department of Evolutionary Biology and Environmental Studies, University of Zurich, Winterthurerstrasse 190, 8057 Zurich, Switzerland; 4 Department of Linguistic and Cultural Evolution (DLCE), Max-Planck Institute for the Science of Human History (MPI-SHH), Kahlaische Str. 10, 07745 Jena, Germany; 5 Palaeo-Research Institute, University of Johannesburg, P.O. Box 524, Auckland Park, 2006 South Africa; 6 SciLifeLab, Uppsala, Sweden

**Keywords:** SNP array, Haplogroup assignment, mtDNA, Africa, HaploGrep

## Abstract

**Background:**

Mitochondrial haplogroup assignment is an important tool for forensics and evolutionary genetics. African populations are known to display a high diversity of mitochondrial haplogroups. In this research we explored mitochondrial haplogroup assignment in African populations using commonly used genome-wide SNP arrays.

**Results:**

We show that, from eight commonly used SNP arrays, two SNP arrays outperform the other arrays when it comes to the correct assignment of African mitochondrial haplogroups. One array enables the recognition of 81% of the African mitochondrial haplogroups from our compiled dataset of full mitochondrial sequences. Other SNP arrays were able to assign 4–62% of the African mitochondrial haplogroups present in our dataset. We also assessed the performance of available software for assigning mitochondrial haplogroups from SNP array data.

**Conclusions:**

These results provide the first cross-checked quantification of mitochondrial haplogroup assignment performance from SNP array data. Mitochondrial haplogroup frequencies inferred from most common SNP arrays used for human population analysis should be considered with caution.

**Supplementary Information:**

The online version contains supplementary material available at 10.1186/s12863-021-01000-2.

## Background

Mitochondrial DNA (mtDNA) is a genetic marker commonly used to study the matrilineal genetic diversity in a population [1]. The mitochondrial genome is circular and spans approximately 16.6 kilobases (kb) [2]. Under ordinary circumstances, the mtDNA is exclusively inherited from the mother, which is referred to as matrilineal inheritance [3].

There is a wide variety of mitochondrial DNA sequences across worldwide human populations. The differences between the various mitochondrial sequences can be used to classify the sequences into phylogenetic clusters called haplogroups. Mitochondrial haplogroups are collections of sequences that have been inherited from the same common ancestor [4]. Therefore, they also share specific single-nucleotide polymorphisms (SNPs) that have accumulated through time.

The haplogroup nomenclature is determined by letters and numbers, adding a digit or letter every time a subdivision into sub-branches is made. The starting letter of each haplogroup does not correspond to the order of branching in the phylogenetic tree, but to the chronological order of discovery. A phylogenetic scheme of the major haplogroup branches and relative nomenclature can be found in available references such as PhyloTree (https://www.phylotree.org/tree/index.htm) [4]. The root of the human mtDNA tree is referred to as “macrohaplogroup L”; as the tree is monophyletic, all known human lineages belong to this macrohaplogroup. It is possible to subdivide macrohaplogroup L into further lineages or subclades: these are named L0, L1, L2, L3, L4, L5 and L6. The mitochondrial haplogroup L3 gave rise to all the mitochondrial haplogroups observed outside Africa: M, N and R (the latter being a subclade of N) and their various subgroups [4]. The rest of the mitochondrial haplogroups (L0, L1, L2, L4, L5, L6 and specific clades of L3) are exclusively found in Africa and will hereafter be referred to as African mitochondrial haplogroups. Most of the human genetic variation is found within Africa, as a consequence of the evolution of our species on this continent before the Out of Africa migration(s): this is found not only in the nuclear genome but also in the mtDNA [5–7].

Mitochondrial haplogroup assignment is of relevance for forensic and evolutionary genetics studies. Haplogroup frequencies vary amongst populations as a result of their history of migration and dispersal. The majority of haplogroups has a phylogeographic relevance, being associated to the region where the haplogroup originated and/or is most commonly found. In early studies, haplogroup assignment was performed by sequencing only the non-coding D-loop region of the mtDNA and/or by typing specific clade-defining restriction fragment length polymorphisms (RFLPs). Nowadays, haplogroup assignment is performed by looking at diagnostic mutations found in the whole mtDNA. When the whole mitochondrial genome is sequenced, the haplogroup assignment is straightforward. For this purpose, many (free) tools for automated haplogroup assignment are available, such as HaploGrep2 [8] and HaploFind [9]. Genome-wide SNP arrays designed for medical and/or population genetics studies often include variant positions from the mitochondrial region. MtDNA SNPs represented on genome-wide SNP arrays could also be used to assign mitochondrial haplogroups, depending on how strategically chosen those variants are. Thus, a dataset generated with SNP arrays could potentially be used not only for analysis of nuclear genetic variation, but also for analysis of mitochondrial haplogroups. This would avoid the extra steps in mtDNA genome sequencing and the relative additional costs and analysis time.

We identify three methods to perform assignment of mitochondrial haplogroups using SNP array data: 1) by constructing a phylogenetic tree with SNPs from mitochondrial sequences of known haplogroups, together with mtDNA SNP sets of unknown haplogroups, and identify the latter according to their placement in the known branches of the phylogenetic tree; 2) by manually searching for known clade-defining mutations; 3) by running software tools to assign haplogroups. Currently, only two software tools can be applied to SNP based genotype data (as opposed to whole mtDNA genome data); namely Hi-MC and HaploGrep2. The Hi-MC method [10] uses a custom panel of 54 mtDNA SNPs (Supplementary Table 5) to assign mitochondrial haplogroups. HaploGrep2 uses a VCF file as input that contains the SNP data [8], and matches the position to the latest PhyloTree reference.

In this research, we assess the ability of commercial SNP arrays to assign mitochondrial haplogroups correctly by comparing the performance of the mtDNA SNP selection characteristic of each array against the performance of full mitochondrial genomes obtained from published data. We restrict our search to African mitochondrial variation to provide a general, broad view on human global mitochondrial variation, and to test if SNP arrays designed specifically with African population diversity in mind show specificity for African haplogroups.

## Results

We assessed the potential of commercial SNP arrays to provide information on mitochondrial haplogroup assignment. We compared their performance to the one obtained by mitochondrial genomes of known haplogroup assignation, retrieved from published data. These comparisons provided us with precision estimates. These estimates show the percentage of haplogroups assigned using the SNP array data that matched the haplogroup reported on the NCBI Genbank. We have done this by comparing phylogenetic trees as well as outcomes based on software tools. We will describe the results from these comparisons separately. We compared the mtDNA SNP selection of eight commercially available arrays, which covered a range of variants from 111 to 522 SNPs (Table 1). Three of these arrays were designed to cover the diversity of populations of African descent.

**Table 1:** SNP arrays investigated in the current study

Corresponding number	SNP array	Version SNP data	Number of mitochondrial SNPs
1	Affymetrix™ Genome-Wide Human SNP Array 6.0	January 2017^a^	411
2	Axiom™ Genome-Wide Human Origins 1 Array	February 2015	256
3	Axiom™ Genome-Wide PanAFR Genotyping Bundle	January 2017^a^	239
4	H3Africa Array	November 2018	260
5	Illumina Infinium Multi-Ethnic AMR/AFR-8	July 2015^a^	373
6	Illumina Infinium Multi-Ethnic Global-8	February 2017^a^	522
7	Illumina Infinium Omni2.5–8	February 2018	116
8	Illumina Infinium Omni5–4	July 2016^a^	111

The eight SNP arrays that are compared based on their ability to assign African haplogroups are listed. The version of the SNP array that was used and the number of mitochondrial SNPs are listed for each SNP array. A (^a^) indicates that this is currently the latest version of the SNP panel. All SNP arrays used the hg19/37 reference genome to refer to SNP positions. The SNP arrays are numbered, and these numbers are used to reference to them in the text

### Phylogenetic trees

We constructed phylogenetic trees based on full mtDNA sequences downloaded from NCBI GenBank. We also constructed trees based only on the SNPs present on various SNP arrays (using hg19/37 reference genome positions) (phylogenetic trees available upon request). We compare haplogroup assignment and major branch topology of these trees. Our downloaded sequences were carefully chosen to represent all major African haplogroup clades up to five digits (Methods). Bootstrapping was applied to all trees, resampling the selected SNPs 500x and measuring how often the same tree topology was observed. Higher values indicate that the topology of the clade is well supported.

**Fig. 1:**
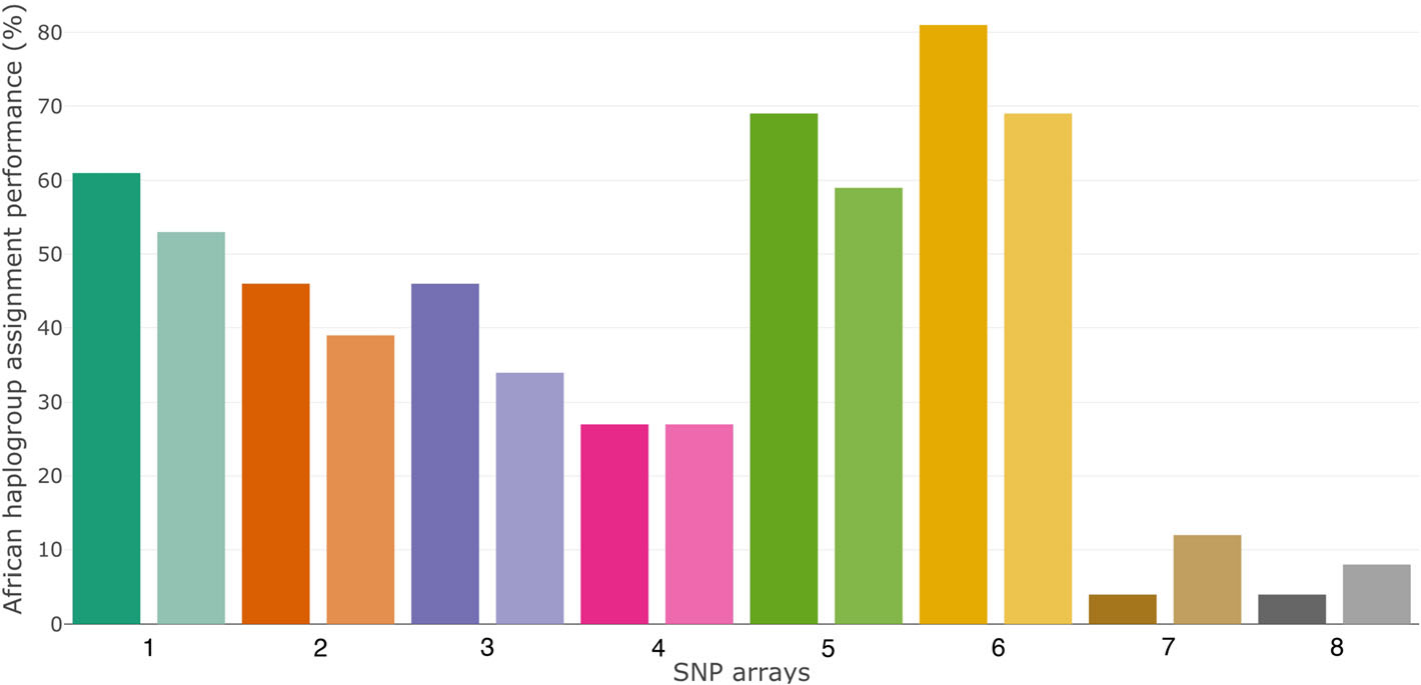
Percentage of assignable mitochondrial haplogroups per SNP array, compared to the full mitochondrial genome. The various SNP arrays are listed on the x-axis. Only clades with a minimum bootstrapping value of 50 were used for the analysis. The two shades represent the level of haplogroup assignment that has been investigated. Darker shades indicate that haplogroups up to the level of three digits (e.g. L0d) have been investigated. Lighter shades indicate that haplogroups up to the level of four digits (e.g. L0d1) have been investigated. Haplogroup assignments from phylogenetic trees based on full mitochondrial genomes were the golden standard. SNP array 5 and 6 show the best performance on African haplogroup assignment

SNP array 5 and 6 showed the best performance when it comes to the assignment of African mitochondrial haplogroups (Fig. 1) (significant for all pairwise comparisons of four-digit values, except for the difference between SNP array 1 and 5, and 1 and 6) (*p*-values in Supplementary Table 1). SNP arrays 5 and 6 were able to assign 59–81% of the African mitochondrial haplogroups (four- and three-digit haplogroups respectively). SNP array 4, a SNP array designed to pick up the wide genetic diversity in African populations [11], was able to assign only 27% of the African mitochondrial haplogroups (for both three- and four-digit haplogroups), significantly worse than SNP array 5 and 6 (Fisher’s exact test; *p* = 7.469e-4, *p* = 7.355e-6). SNP array 7 and 8 were the worst performers when it comes to the assignment of African mitochondrial haplogroups. Their mitochondrial haplogroup assignment in African populations ranged from 4 to 12% at most (three- and four-digit haplogroups respectively). All the other SNP arrays performed moderately when it comes to the assignment of African mitochondrial haplogroups, with efficiencies varying from 34 to 62%.

The four-digit haplogroup assignment score was higher than the three-digit haplogroup assignment score for SNP array 7 and 8. This can be due to the lack of haplogroup defining SNPs in the array at the level of the three-digit haplogroups, but not a lack of haplogroup defining SNPs at the level of the four-digit haplogroups. In this way, major haplogroups can misleadingly be represented as not monophyletic, while their subhaplogroups are represented as monophyletic in the phylogenetic trees based on SNPs. Thus, the fact that two subhaplogroups are monophyletic does not necessarily make their overarching major haplogroup monophyletic as well. This is dependent on the SNPs incorporated on the SNP array. This phenomenon can result in a higher haplogroup assignment score for the mentioned four-digit haplogroups than for the three-digit haplogroup, with the three-digit haplogroup assignment score lower than the four-digit one.

**Fig. 2:**
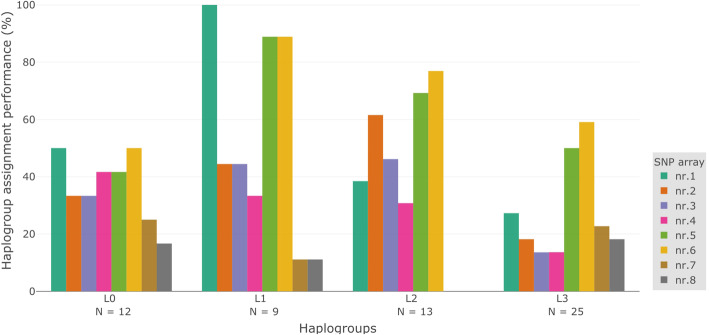
L0-L3 haplogroup assignment performance for eight different SNP arrays. The percentage of haplogroups that could be assigned compared to the full mitochondrial genome is shown for L0-L3. Only clades with a minimum bootstrapping value of 50 were used for the analysis. The different colours indicate the eight SNP arrays. When interested in a specific African haplogroup or a population carrying a specific haplogroup in high frequency, this SNP array analysis can guide researchers in assessing if mitochondrial haplogroup assignment using the particular SNP array will be informative. Up to four-digit haplogroups have been investigated (for example L0, L0d, L0d1). The numbers underneath each haplogroup indicate on how many sequences the analysis for that haplogroup is based

We further examined the mitochondrial haplogroup assignment of different SNP arrays by looking at the specific L0-L3 mitochondrial haplogroups individually. We analysed how well the eight different SNP arrays could assign each of the African haplogroups L0-L3 (Fig. 2). We focused only on the most represented haplogroups, and thus only show results for the less common L4, L5 and L6 in the Supplementary materials (Additional file 1; Supplementary Fig. 3). We observed that most of the L0-L3 mitochondrial haplogroups were assigned best when using SNP array 5 or 6 (Fig. 2). On average, the mitochondrial haplogroups L0, L1 and L2 were assigned best using the SNP arrays we have investigated. Their haplogroup assignment efficiency was 36.5, 52.8 and 40.4% respectively. Mitochondrial haplogroup L3 was least precisely assigned, having an average assignment percentage of 27.8%. The haplogroup assignment of SNP array 7 and 8 is worst for most haplogroups, but especially for haplogroup L2, where they haplogroup assignment efficiency for both arrays is 0%. Interestingly, the performance in assigning haplogroup L0 is equally low (less than 50%) for all the investigated SNP arrays, but SNP arrays 5 and 6 give a better performance in assigning haplogroup L1 and L3. SNP array 1 also provides an excellent performance, but only for haplogroup L1.

We have also investigated mitochondrial haplogroup assignment using trees without a cut-off bootstrapping value (Additional file 1; Supplementary Fig. 1 and 2). Without having a cut-off value, all clades are considered equally reliable. The average differences between results based on a tree with and without bootstrapping values are 12.82% ± 5.15 and 13.29% ± 4.45 (three- and four-digit haplogroups respectively), where the value for the tree that takes into account the bootstrapping values was always lower. In general, these trends were true for all the SNP arrays.

We observe a high positive correlation between the number of mitochondrial SNPs typed on the investigated SNP arrays and the mitochondrial haplogroup assignment performance through phylogenetic trees (Pearson’s correlation coefficient: 0.9698051, *p* = 6.728 · 10^− 5^ for 4-digit haplogroups and Pearson’s correlation coefficient: 0.9470996, *p* = 3.556 · 10^− 4^ for 3-digit haplogroups). No statistical difference can be observed between the correlation for the performance on 3-digit and 4-digit haplogroups (Fisher’s z = − 0.4525, *p*-value = 0.6509, calculated with the cocor package [12]). In general, the more mitochondrial SNPs incorporated on the SNP array, the better the mitochondrial haplogroup assignment with the phylogenetic trees can be performed. This is true in particular for the lowest performance arrays, number 7 and 8, which contain only 116 and 111 mtDNA variants. This correlation does not hold for all arrays: the second-best performing array, SNP array 5, has only 373 mtDNA variants, but performs better than SNP array 1, which includes 411 mtDNA variants.

### Haplogroup assignment software performance

Two software tools can be applied to SNP based data (as opposed to whole mtDNA genome data); namely Hi-MC and HaploGrep2. Hi-MC uses a custom panel of 54 mtDNA SNPs to assign mitochondrial haplogroups (Supplementary Table 5) [10]. The Hi-MC GitHub page (https://github.com/vserch/himc, 28-03-2021) states that haplogroup assignment will not be accurate if more than a few of these SNPs are missing. Thus, the Hi-MC method can only be used for haplogroup assignment if the SNPs from the Hi-MC panel are incorporated in the SNP array. Comparing the SNPs from the Hi-MC panel to the SNPs from the eight different SNP arrays showed too little overlap; 21 up to 53 SNPs of the Hi-MC panel were missing (Supplementary Table 5). Thus, typing individuals on one of the eight investigated SNP arrays would not provide enough information for the Hi-MC panel to determine the mitochondrial haplogroups with high precision. Therefore, the haplogroup determination using Hi-MC was not investigated in the current study.

**Fig. 3:**
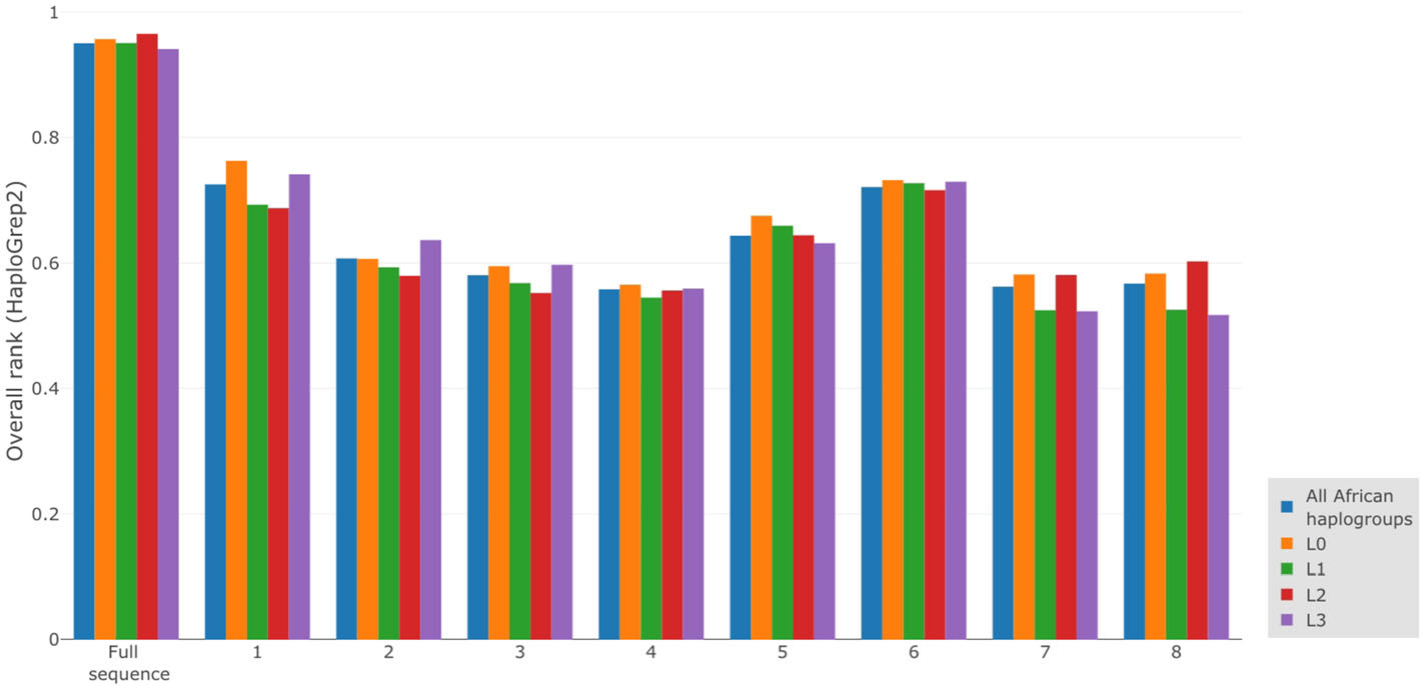
Average haplogroup assignment scores (HaploGrep2). The haplogroup assignment scores from HaploGrep2 were averaged and are shown here for the full mitochondrial sequences as well as each of the individual SNP arrays. The different colours indicate the different haplogroups. The other African haplogroups (L4-L6) are not shown here because of their low sample size

We also assessed the ability of commercial SNP arrays to assign mitochondrial haplogroups correctly by looking at HaploGrep2 outcomes. Firstly, the haplogroup assignment with HaploGrep2 using full mitochondrial genomes was compared with the haplogroup assignment of HaploGrep2 using only SNP array data. HaploGrep2 outputs an “overall rank” for the haplogroup assignment (Fig. 3). The higher the overall rank, the more certain the haplogroup assignment. Whereas the average haplogroup assignment “rank” for the full sequences is 0.95, the average haplogroup assignment “ranks” for the SNP arrays are much lower (ranging from 0.56–0.76).

**Fig. 4:**
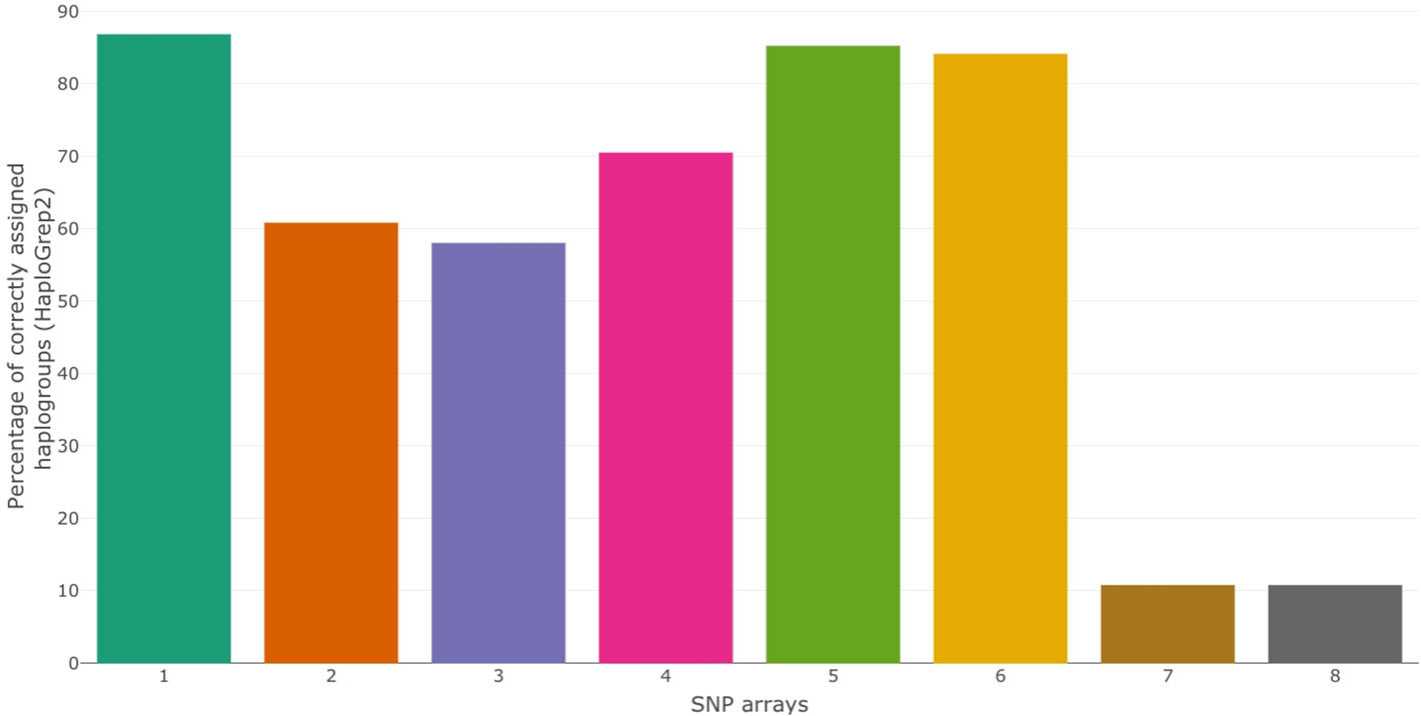
The percentage of correctly assigned African haplogroups by HaploGrep2, using only SNP array data. The percentage of African haplogroups that were correctly assigned by HaploGrep2 using only the SNPs typed on that SNP array is shown. The golden standard is the haplogroup reported in the NCBI GenBank. This analysis does not take into account the haplogroup rank, nor does it take into account the level of haplogroup assignment; whether L0 or L0a2a is assigned, makes no difference for this analysis

We also compared the haplogroup that was assigned by HaploGrep2 to the actual haplogroup (reported in the NCBI GenBank) for each sample. Here, we report the percentage of correctly assigned African haplogroups by HaploGrep2, using only SNP array data (Fig. 4). SNP array 7 and 8 show very low haplogroup assignment percentages. Leaving these two SNP arrays aside, we generally observe moderate to good haplogroup assignment percentages (58–86%). We observe similar results when using HaploGrep2 for the full genomes as a golden standard, instead of the haplogroup reported in the NCBI GenBank. Here, SNP array 7 and 8 also show low haplogroup assignment percentages. When we leave them out, we observe haplogroup assignment percentages ranging from 64 to 96% (Additional file 1; Supplementary Fig. 4).

Imputation can also be considered to increase the number of mitochondrial variants available based on SNP array data. MitoImpute is a tool designed to impute missing data for the mitochondrial genome [13], and could also be utilized for SNP array data. We used MitoImpute to impute variants for the SNP array data of the 211 individuals. Afterwards, haplogroups were assigned again using HaploGrep2, and haplogroup assignments were compared with HaploGrep2 assignments of non-imputed SNP array data. We find that the imputation decreased the haplogroup assignment performance (13–90%) for the African haplogroups. This decrease in performance could be due to the lack of coverage for African haplogroups at this point; future versions of the tool might be improved with the addition of broader reference panels.

## Discussion

Despite recent advances in genome-wide studies, mitochondrial haplogroup assignment is still of relevance for forensic and evolutionary genetics. The feasibility of mitochondrial haplogroup assignment from SNP arrays has long been debated and depends heavily on the SNPs incorporated on a SNP array. In this study, we focus on African haplogroup diversity to include the major branches of global mtDNA diversity. We show that across the tests performed, SNP array 5 and 6 have the best performance. Moreover, we provide detailed information about which SNP arrays can be used best when interested in a specific African haplogroup.

Interestingly, we observe the same general trend for the performance among the different SNP arrays, independent on the haplogroup assignment method (inferring haplogroups from the position in the phylogenetic tree (Fig. 1) and HaploGrep2 (Fig. 3)). In both the analysis based on comparing phylogenetic trees and the analysis based on HaploGrep2, we see better haplogroup assignments scores/ranks for the SNP array 5 and 6. The haplogroup assignments by HaploGrep2 in the best performing SNP arrays (SNP array 1, 5 and 6) reach more than 80% precision (Fig. 4). While the high number of correctly assigned haplogroups might be relevant, the percentage of wrong assignments makes results of population haplogroup compositions not comparable to results obtained from mtDNA genome sequencing or direct typing of diagnostic SNPs.

Although we observe a high positive correlation between the number of mitochondrial SNPs typed on the investigated SNP arrays and the mitochondrial haplogroup assignment performance through phylogenetic trees, it is worth noting that the results are not solely dependent on the total number of mtDNA variants included in the array. The informativeness of the variants also plays a role. For example, SNP array 5 (373 mtDNA SNPs) shows a performance superior to SNP array 1 (411 mtDNA SNPs) in our tested samples (Fig. 1). SNP array 5 was designed specifically to explore genetic diversity in Hispanic and African-American populations. We would also like to point out that the SNP array designed specifically with African population diversity in mind, SNP array 4, does not show the best performance for mitochondrial haplogroup classification. The inclusion of more SNPs representing African mitochondrial diversity would benefit this SNP array, and its aims of covering African population diversity.

Caution should always be taken when interpreting mitochondrial haplogroups inferred from SNP array data. In this study, we have shown that the extent of incorrect haplogroup assignments varies between the different SNP arrays, and also depends on the method used to assign mitochondrial haplogroups. Our results provide guidance on to which degree mtDNA haplogroup assignment can be a useful and informative pursuit when analysing a specific SNP array dataset. Additionally, for SNP arrays not included in our analyses, our study outlines a pipeline to assess the level of performance of SNP array data in assigning mtDNA haplogroups. Until more representative SNPs are included on SNP arrays, full mitochondrial genomes remain the most informative source for mitochondrial haplogroup assignment. As more and more platforms are offering ways to sequence full mitochondrial genomes (e.g. PacBio, Oxford Nanopore), it becomes easier and cheaper to generate full mitochondrial data. This will provide great opportunities for forensic and evolutionary genetics in the future.

## Conclusions

We show the level of performance of various SNP arrays for the assignment of African mitochondrial haplogroups using phylogenetic trees based on SNP array data, as well as the level of performance of HaploGrep2 in assigning African mitochondrial haplogroups for SNP array data. In general, SNP array 5 and 6 perform best. The testing of various SNP arrays presented in this study regarding their ability to assign mitochondrial haplogroups correctly will help researchers to decide whether they should include mitochondrial haplogroup assignment in their research based on SNP arrays.

## Methods

We compared SNP arrays with regard to their ability to correctly assign mitochondrial haplogroups from Africa by 1) comparing phylogenetic trees and 2) running HaploGrep2. To do this, 211 full mitochondrial sequences (Additional file 2; Supplementary Table 3) were downloaded from the NCBI GenBank (https://www.ncbi.nlm.nih.gov/), one from each five-digit African haplogroup (for example; L0b1a) and two for each one-digit non-African haplogroup (example; H). This was done according to the haplogroup classification reported in PhyloTree 2016 (https://www.phylotree.org/tree/index.htm) [4]. In comparison, more sequences were taken for the African haplogroups in order to focus on African haplogroups specifically. The downloaded sequences were aligned to the revised Cambridge Reference Sequence (rCRS, NCBI GenBank Accession Number NC_012920) using MUSCLE alignment [14]. Where gaps were created in the reference sequence due to the alignment, they were deleted so as to maintain the reference genome nucleotide numbering. Eight SNP arrays were chosen for the comparison based on the presumed applicability in African populations (Table 1). Three of the chosen arrays (number 3, 4 and 5) are designed with a focus on variants present in populations of African ancestry. For all the SNP arrays, mtDNA SNP positions (genome built: GRCh37, mtDNA: rCRS) were obtained (Supplementary Fig. 4) and we extracted the bases corresponding to these SNP positions for all 211 mitochondrial sequences.

### Comparing phylogenetic trees

Phylogenetic trees were built for every SNP array, using the extracted bases at the SNP positions (Supplementary Table 4) only, using the neighbour-joining method in MEGA (uniform rates, Maximum Composite Likelihood). Bootstrapping (500 replicates) has been applied to the trees [15]. The eight SNP arrays were compared for their mitochondrial DNA markers with regard to their ability to assign the African mitochondrial haplogroups. A haplogroup was considered “assignable” if all or all but one of its subhaplogroups clustered together under one branch in the tree (minimum bootstrap value: 50), with no more than one other subhaplogroup that should not belong to this branch. Haplogroup assignment in a tree based on full mitochondrial sequences was used as the golden standard. The ability to assign the African haplogroups was compared for haplogroups up to three (for example L0d) and four digits (for example L0d1). Deeper clustering was not considered since only one sequence for each five-digit haplogroup was included in the study design. Plots were created using the ggplot2 package in R. The four-digit haplogroup assignment efficiencies of the eight different SNP arrays were compared using the Fisher’s exact test. *P*-values for pairwise comparisons were calculated.

### HaploGrep2

We also compared the haplogroup assignment of HaploGrep2 using full mitochondrial genomes with the haplogroup assignment of HaploGrep2 using only SNP array data. First, HaploGrep2 was run using the 211 aligned full mitochondrial sequences. After this, we converted a multi-aligned FASTA file into a VCF file using SNP-sites software (snp-sites -v -c [−o *output_filename*] [*input file*]) [16] and extracted array-specific positions with vcftools (vcftools [−-vcf *file 1.vcf*] [−-positions-overlap *file 2.txt]* --out *outputname --recode*) [17] (Supplementary Section 1). This created eight VCF files, containing only the positions with corresponding bases for the 211 sequences. These VCF files were used as input for HaploGrep2. HaploGrep2 outputs mtDNA haplogroups, as well as an “overall rank” for the haplogroup assignment. These overall ranks were compared for full mitochondrial sequences and for the different SNP arrays.

Moreover, for each sample and for every SNP array, we compared the haplogroup that was assigned by HaploGrep2 to the haplogroup reported in the NCBI GenBank and the haplogroup outputted by HaploGrep2 when using full genomes.

### MitoImpute

Imputation of mitochondrial variants for SNP array data of the 211 individuals was done using MitoImpute [13]. Settings: REFAF: 0.001, INFOCUT: 0, ITER: 2, BURNIN: 1, KHAP: 1000. Afterwards, haplogroups were assigned using HaploGrep2 and haplogroup assignments were compared with HaploGrep2 assignments of non-imputed SNP array data for all African haplogroups.

## Supplementary Information

**Additional file 1 Table S1.** *P*-values of comparisons SNP array performances. **Table S2.** P-values of comparisons SNP array performances (no bootstrapping performed). **Fig. S1.** Percentage of assignable mitochondrial haplogroups compared to full mitochondrial genome per SNP array (no bootstrapping). **Fig. S2.** L0-L6 haplogroup assignment performance for eight different SNP arrays (no bootstrapping). **Fig. S3.** L0-L6 haplogroup assignment performance for eight different SNP arrays (bootstrapping applied). **Fig. S4.** The percentage of correctly assigned African haplogroups by HaploGrep2, using only SNP array data. Supplementary Section 1. Here, we provide the script used to create VCF files from aligned FASTA files.

**Additional file 2 Table S3.** The 211 sequences used for building of phylogenetic trees. **Table S4.** Mitochondrial SNP positions incorporated on the eight different SNP arrays. **Table S5.** Hi-MC panel SNPs and their overlap with the eight different SNP arrays.

## Data Availability

No new data was generated during the study. The datasets analysed during the current study was downloaded from NCBI Genbank repository, (http://www.ncbi.nlm.nih.gov). The NCBI GenBank accession numbers for all individuals analysed in the current study can be found in Supplementary Table 3.

## Abbreviations

kb: Kilobases
mtDNA: Mitochondrial deoxyribonucleic acid
rCRS: Revised Cambridge Reference Sequence
RFLPs: Restriction fragment length polymorphisms
SNP: Single-nucleotide polymorphism
