# Supplementary material for: The performance of common SNP arrays in assigning African mitochondrial haplogroups
Source: BMC Genom Data. 2021 Oct 21;22:43. doi: 10.1186/s12863-021-01000-2 (PMC8532338; doi:10.1186/s12863-021-01000-2)
Supplement: Supplementary file 1 — Additional file 1 Table S1. P-values of comparisons SNP array performances. Table S2. P-values of comparisons SNP array performances (no bootstrapping performed). Fig. S1. Percentage of assignable mitochondrial haplogroups compared to full mitochondrial genome per SNP array (no bootstrapping). Fig. S2. L0-L6 haplogroup assignment performance for eight different SNP arrays (no bootstrapping). Fig. S3. L0-L6 haplogroup assignment performance for eight different SNP arrays (bootstrapping applied). Fig. S4. The percentage of correctly assigned African haplogroups by HaploGrep2, using only SNP array data. Supplementary Section 1. Here, we provide the script used to create VCF files from aligned FASTA files. [file 12863_2021_1000_MOESM1_ESM.zip › Additional file 1(14-09-2021).docx]

# Supplementary Material for

# The performance of common SNP arrays in assigning African mitochondrial haplogroups

Imke Lankheet, Mário Vicente, Chiara Barbieri, Carina Schlebusch

Corresponding author: Carina Schlebusch (carina.schlebusch@ebc.uu.se)

**The Supplementary Material includes:**

Supplementary Table 1. P-values of comparisons SNP array performances.

Supplementary Table 2. P-values of comparisons SNP array performances (no bootstrapping performed).

Supplementary Table 3. The 211 sequences used for building of phylogenetic trees.

Supplementary Table 4. Mitochondrial SNP positions incorporated on the eight different SNP arrays.

Supplementary Table 5. Hi-MC panel SNPs and their overlap with the eight different SNP arrays.

Supplementary Figure 1. Percentage of assignable mitochondrial haplogroups compared to full mitochondrial genome per SNP array (no bootstrapping).

Supplementary Figure 2. L0-L6 haplogroup assignment performance for eight different SNP arrays (no bootstrapping).

Supplementary Figure 3. L0-L6 haplogroup assignment performance for eight different SNP arrays (bootstrapping applied).

Supplementary Figure 4. The percentage of correctly assigned African haplogroups by HaploGrep2, using only SNP array data.

Supplementary Section 1. Script used to create VCF files from aligned FASTA files.

***Supplementary Table 1. P-values of comparisons SNP array performances.***

| **SNP array 2** | 0.1957 |  |  |  | |  |  |
| --- | --- | --- | --- | --- | --- | --- | --- |
| **SNP array 3** | 0.0626 | 0.7023 |  |  |  |  |  |
| **SNP array 4** | 0.0081* | 0.2402 | 0.549 |  |  |  |  |
| **SNP array 5** | 0.5782 | 0.0424* | 0.0095* | 7.469e-4* |  |  |  |
| **SNP array 6** | 0.0889 | 0.0016* | 2.003e-4* | 7.355e-6* | 0.3364 |  |  |
| **SNP array 7** | 3.398e-6* | 0.0013* | 0.0078* | 0.06147 | 9.135e-8* | 1.485e-10* |  |
| **SNP array 8** | 2.267e-7* | 1.607e-4* | 0.0013* | 0.01467* | 4.543e-9* | 4.85e-12* | 0.762 |
|  | **SNP array 1** | **SNP array 2** | **SNP array 3** | **SNP array 4** | **SNP array 5** | **SNP array 6** | **SNP array 7** |

*The four-digit haplogroup assignment performances of the eight different SNP arrays were compared using the Fisher’s exact test. The p-values for the pairwise comparisons are shown in this table. Significant differences with p<0.05 are indicated with a star (*).*

***Supplementary Table 2. P-values of comparisons SNP array performances (no bootstrapping performed).***

| **SNP array 2** | 0.0037* |  |  |  |  |  |  |
| --- | --- | --- | --- | --- | --- | --- | --- |
| **SNP array 3** | 0.0021* | 1 |  |  |  |  |  |
| **SNP array 4** | 3.064e-4* | 0.5874 | 0.7168 |  |  |  |  |
| **SNP array 5** | 0.4083 | 9.659e-5* | 4.654e-5* | 4.335e-6* |  |  |  |
| **SNP array 6** | 0.2037 | 1.526e-5* | 6.896e-6* | 5.311e-7* | 0.8209 |  |  |
| **SNP array 7** | 2.611e-6* | 0.0953 | 0.1365 | 0.3448 | 1.477e-8* | 1.244e-9* |  |
| **SNP array 8** | 3.838e-7* | 0.0392* | 0.0596 | 0.18 | 1.599e-*9 | 1.19e-10* | 0.8412 |
|  | **SNP array 1** | **SNP array 2** | **SNP array 3** | **SNP array 4** | **SNP array 5** | **SNP array 6** | **SNP array 7** |

*The four-digit haplogroup assignment performances of the eight different SNP arrays were compared using the Fisher’s exact test. The p-values for the pairwise comparisons are shown in this table. Significant differences with p<0.05 are indicated with a star (*).*

***Supplementary Figure 1. Percentage of assignable mitochondrial haplogroups compared to full mitochondrial genome per SNP array (no bootstrapping).*** *The various SNP arrays are listed on the x-axis. The two shades represent the level of haplogroup assignment that has been investigated. Darker shades indicate that haplogroups up to the level of three digits (e.g. L0d) have been investigated. Lighter shades indicate that haplogroups up to the level of four digits (L0d1) have been investigated. SNP array 5 and 6 show the best performance on African haplogroup assignment. This figure is based on phylogenetic trees without bootstrapping values.*

***Supplementary Figure 2.*** ***L0-L6 haplogroup assignment performance for eight different SNP arrays (no bootstrapping).*** *The percentage of haplogroups that could be assigned compared to the full mitochondrial genome is shown for L0-L6. The different colours indicate the eight SNP arrays. When interested in a specific African haplogroup or a population carrying a specific haplogroup in high frequency, this SNP array analysis can guide researchers in assessing if mitochondrial haplogroup assignment using the particular SNP array will be useful. Up to four-digit haplogroups have been investigated (for example L0, L0d, L0d1). The numbers underneath each haplogroup indicate on how many sequences the analysis for that haplogroup is based. This figure is based on phylogenetic trees without bootstrapping values.*

***Supplementary Figure 3.*** ***L0-L6 haplogroup assignment performance for eight different SNP arrays (bootstrapping applied).*** *The percentage of haplogroups that could be assigned compared to the full mitochondrial genome is shown for L0-L6. Only clades with a minimum bootstrapping value of 50 were used for the analysis. The different colours indicate the eight SNP arrays. When interested in a specific African haplogroup or a population carrying a specific haplogroup in high frequency, this SNP array analysis can guide researchers in assessing if mitochondrial haplogroup assignment using the particular SNP array will be useful. Up to four-digit haplogroups have been investigated (for example L0, L0d, L0d1). The numbers underneath each haplogroup indicate on how many sequences the analysis for that haplogroup is based.*

***Supplementary Figure 4. The percentage of correctly assigned African haplogroups by HaploGrep2, using only SNP array data.*** *The percentage of African haplogroups that were correctly assigned by HaploGrep2 using only the SNPs typed on that SNP array is shown. The golden standard is the haplogroup output for the full genomes using HaploGrep2. This analysis does not take into account the haplogroup rank, nor does it take into account the level of haplogroup assignment; whether L0 or L0a2a is assigned, makes no difference for this analysis.*

**Supplementary Section 1**

Here, we provide the script used to create VCF files from aligned FASTA files.

# Downloading conda:

curl -O https://repo.anaconda.com/miniconda/Miniconda3-latest-Linux-x86_64.sh

sh Miniconda3-latest-Linux-x86_64.sh

# Installing snp-sites

conda config --add channels conda-forge

conda config --add channels defaults

conda config --add channels r

conda config --add channels bioconda

conda install snp-sites

# Running snp-sites to make a VCF file from a multi-aligned fasta file

snp-sites -v -c [-o output_filename] [input file]

# Filtering the VCF file to the SNPs typed on the SNP array

module load bioinfo-tools

module load vcftools

vcftools [--vcf file1.vcf] [--positions-overlap file2.txt] --out outputname --recode
