# Supplementary figures and images for: The performance of common SNP arrays in assigning African mitochondrial haplogroups
Source: BMC Genom Data. 2021 Oct 21;22:43. doi: 10.1186/s12863-021-01000-2 (PMC8532338; doi:10.1186/s12863-021-01000-2)

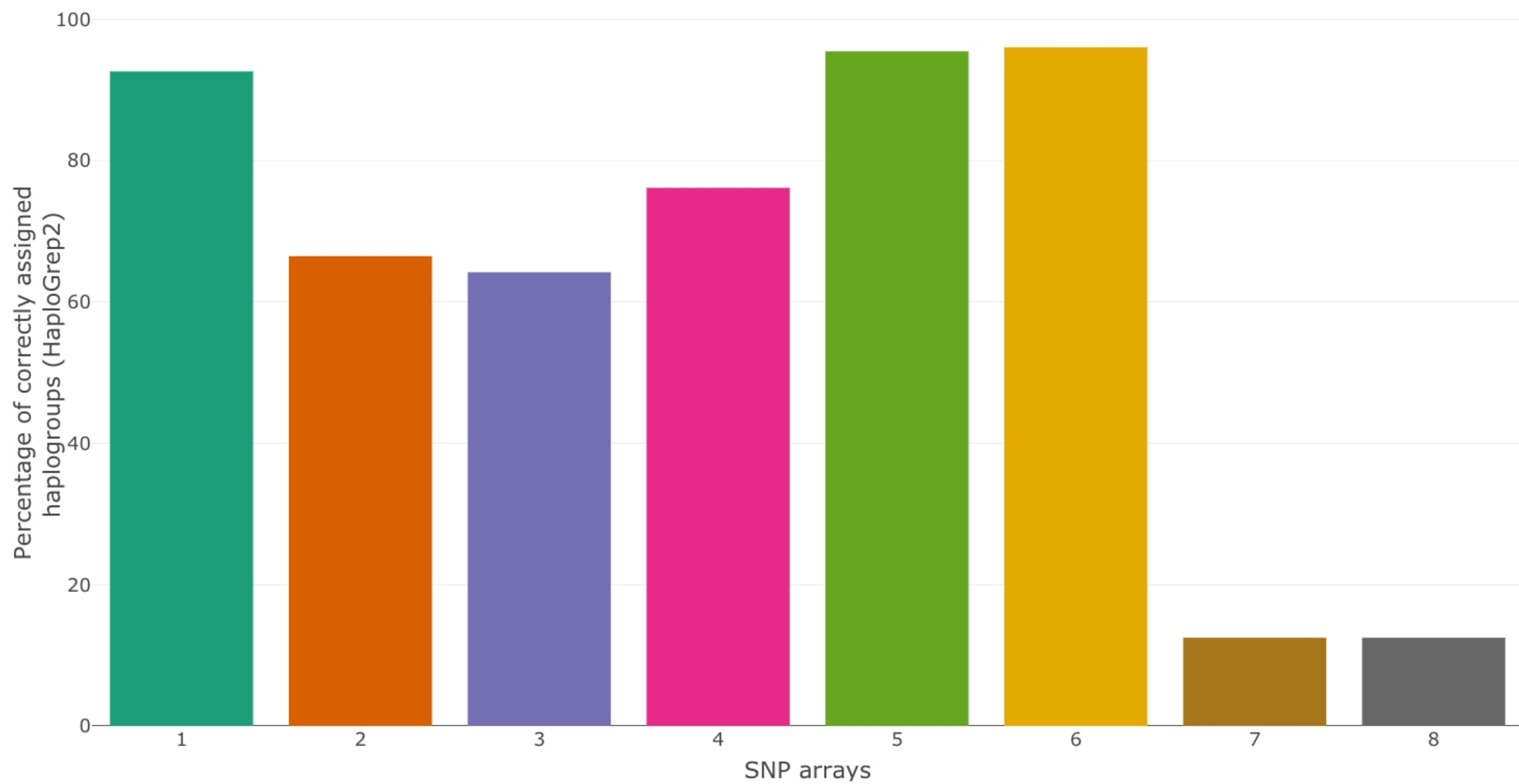

Supplement: Supplementary file 1 — Additional file 1 Table S1. P-values of comparisons SNP array performances. Table S2. P-values of comparisons SNP array performances (no bootstrapping performed). Fig. S1. Percentage of assignable mitochondrial haplogroups compared to full mitochondrial genome per SNP array (no bootstrapping). Fig. S2. L0-L6 haplogroup assignment performance for eight different SNP arrays (no bootstrapping). Fig. S3. L0-L6 haplogroup assignment performance for eight different SNP arrays (bootstrapping applied). Fig. S4. The percentage of correctly assigned African haplogroups by HaploGrep2, using only SNP array data. Supplementary Section 1. Here, we provide the script used to create VCF files from aligned FASTA files. [file 12863_2021_1000_MOESM1_ESM.zip › SupplementaryFigure4.pdf]

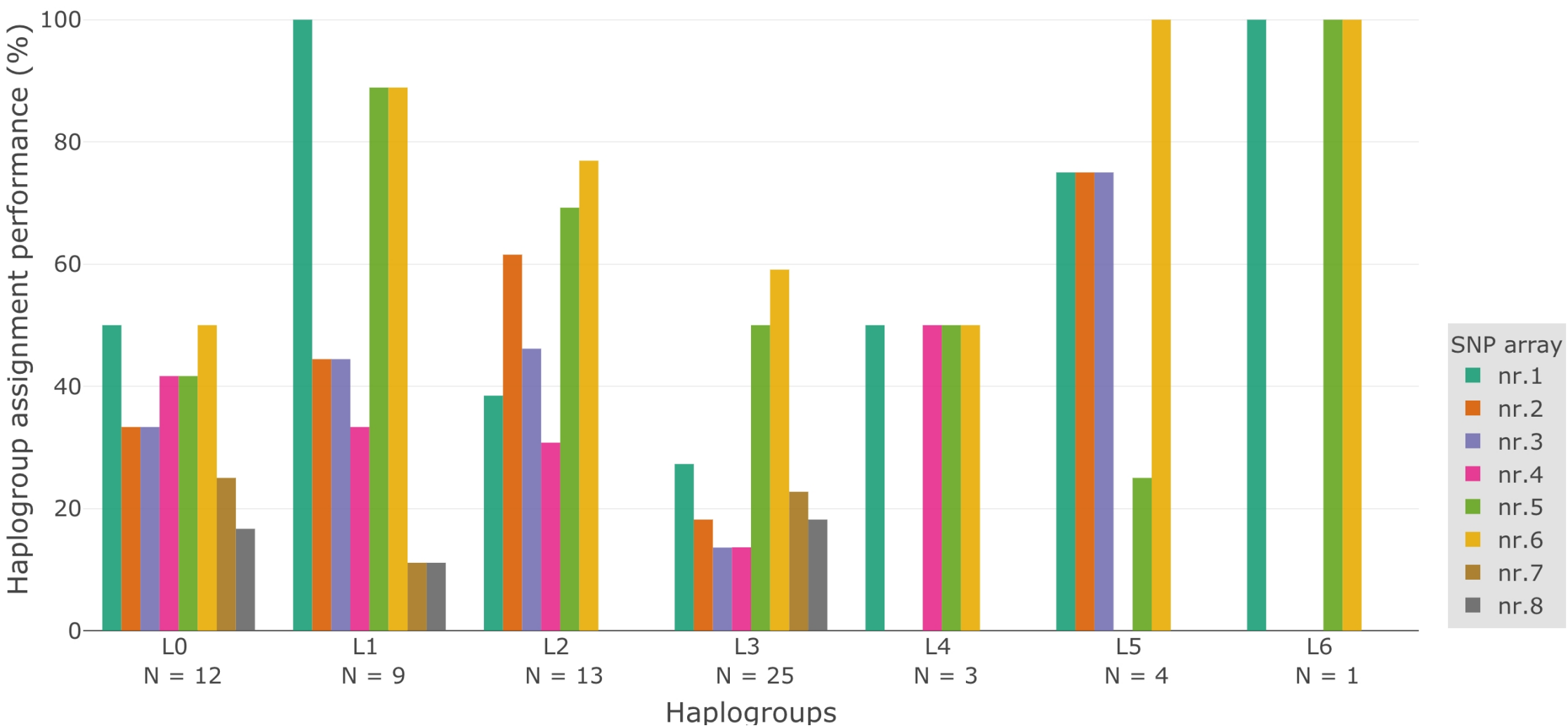

Supplement: Supplementary file 1 — Additional file 1 Table S1. P-values of comparisons SNP array performances. Table S2. P-values of comparisons SNP array performances (no bootstrapping performed). Fig. S1. Percentage of assignable mitochondrial haplogroups compared to full mitochondrial genome per SNP array (no bootstrapping). Fig. S2. L0-L6 haplogroup assignment performance for eight different SNP arrays (no bootstrapping). Fig. S3. L0-L6 haplogroup assignment performance for eight different SNP arrays (bootstrapping applied). Fig. S4. The percentage of correctly assigned African haplogroups by HaploGrep2, using only SNP array data. Supplementary Section 1. Here, we provide the script used to create VCF files from aligned FASTA files. [file 12863_2021_1000_MOESM1_ESM.zip › SupplementaryFigure3.pdf]

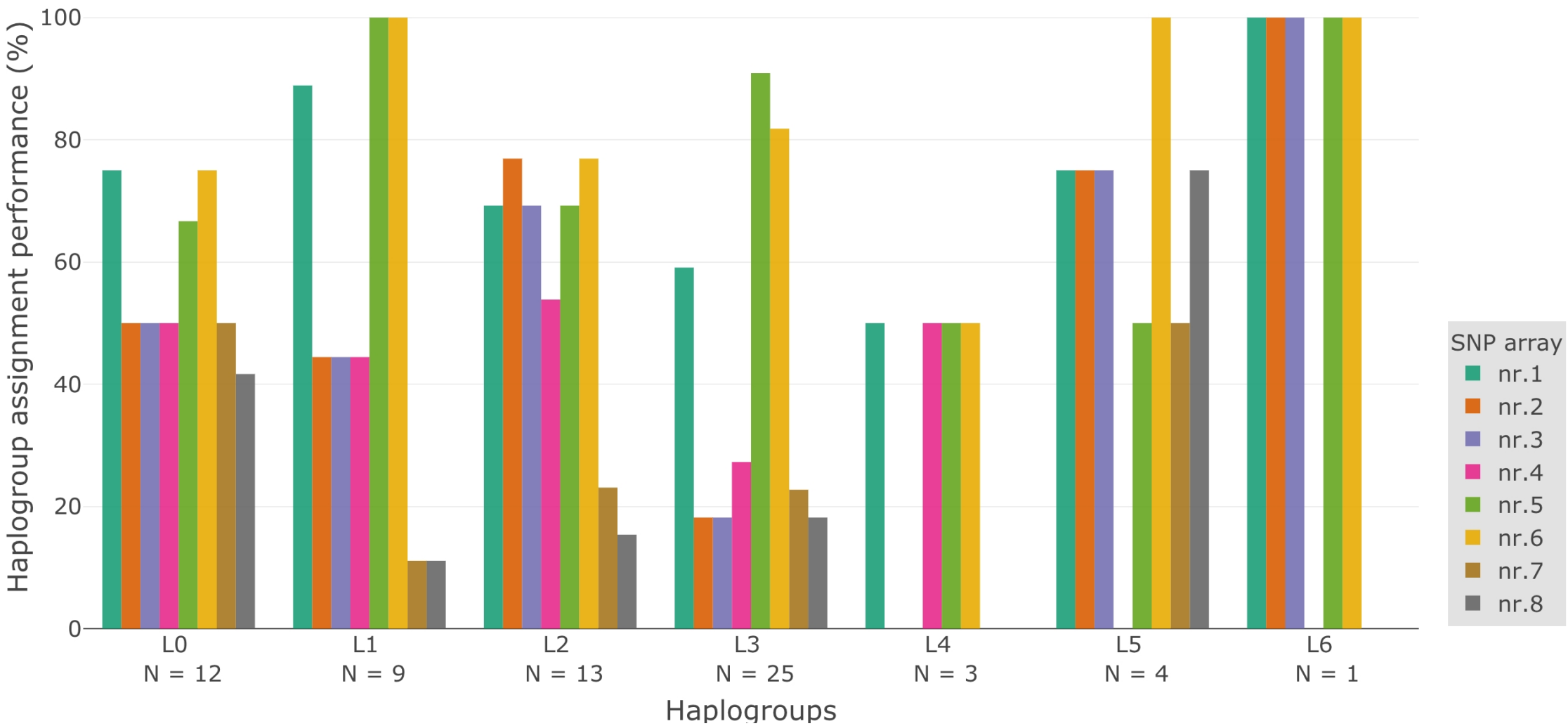

Supplement: Supplementary file 1 — Additional file 1 Table S1. P-values of comparisons SNP array performances. Table S2. P-values of comparisons SNP array performances (no bootstrapping performed). Fig. S1. Percentage of assignable mitochondrial haplogroups compared to full mitochondrial genome per SNP array (no bootstrapping). Fig. S2. L0-L6 haplogroup assignment performance for eight different SNP arrays (no bootstrapping). Fig. S3. L0-L6 haplogroup assignment performance for eight different SNP arrays (bootstrapping applied). Fig. S4. The percentage of correctly assigned African haplogroups by HaploGrep2, using only SNP array data. Supplementary Section 1. Here, we provide the script used to create VCF files from aligned FASTA files. [file 12863_2021_1000_MOESM1_ESM.zip › SupplementaryFigure2.pdf]

African haplogroup assignment performance (%)

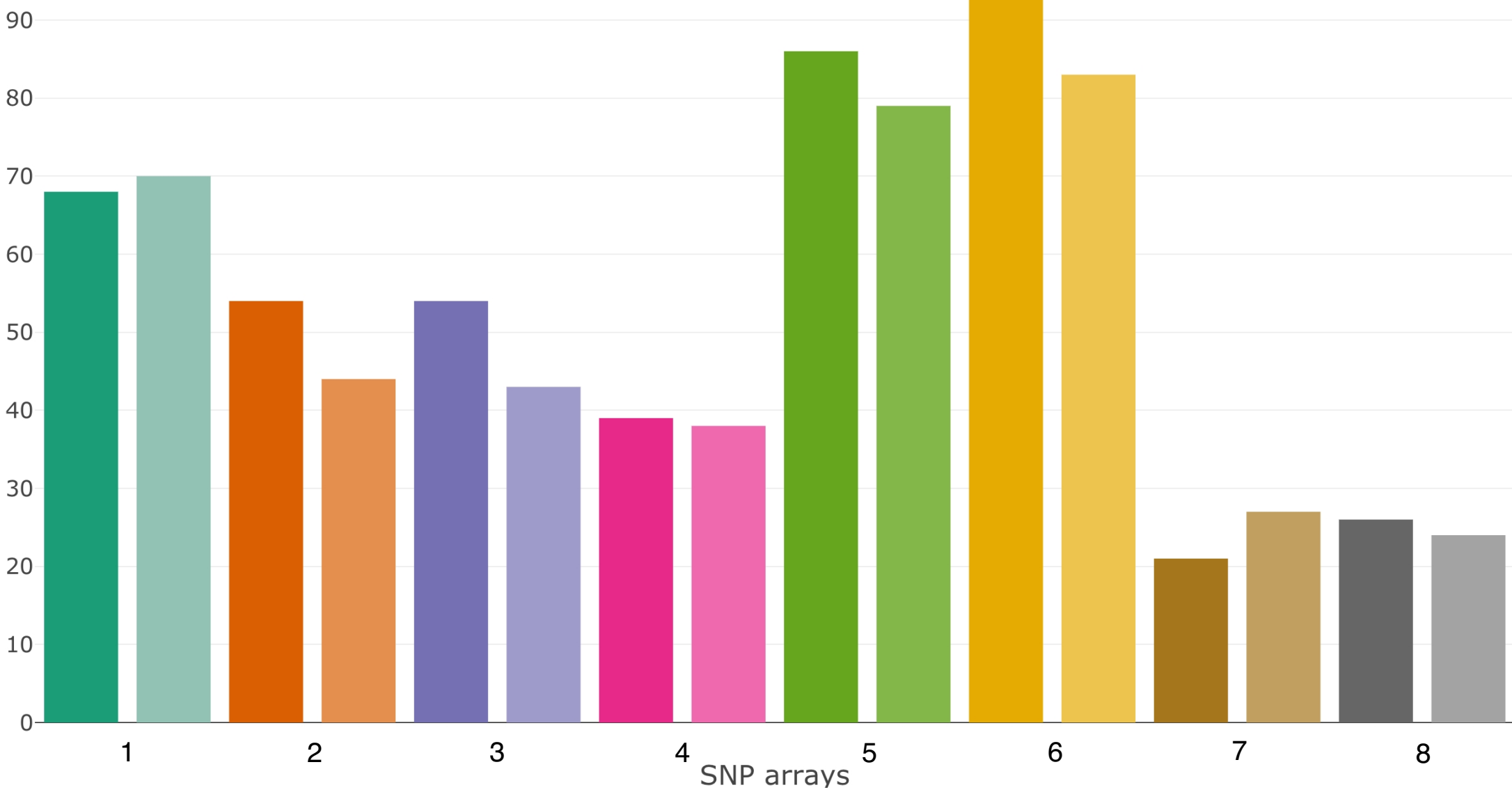

Supplement: Supplementary file 1 — Additional file 1 Table S1. P-values of comparisons SNP array performances. Table S2. P-values of comparisons SNP array performances (no bootstrapping performed). Fig. S1. Percentage of assignable mitochondrial haplogroups compared to full mitochondrial genome per SNP array (no bootstrapping). Fig. S2. L0-L6 haplogroup assignment performance for eight different SNP arrays (no bootstrapping). Fig. S3. L0-L6 haplogroup assignment performance for eight different SNP arrays (bootstrapping applied). Fig. S4. The percentage of correctly assigned African haplogroups by HaploGrep2, using only SNP array data. Supplementary Section 1. Here, we provide the script used to create VCF files from aligned FASTA files. [file 12863_2021_1000_MOESM1_ESM.zip › SupplementaryFigure1.pdf]
